# Shadow Detection Based on Regions of Light Sources for Object Extraction in Nighttime Video

**DOI:** 10.3390/s17030659

**Published:** 2017-03-22

**Authors:** Gil-beom Lee, Myeong-jin Lee, Woo-Kyung Lee, Joo-heon Park, Tae-Hwan Kim

**Affiliations:** 1 Telecommunications Technology Association, 47 Bundang-ro, Bundang-gu, Seongnam-si, Gyeonggi-do 13591, Korea; lgbch2@tta.or.kr; 2 School of Electronics and Information Engineering, Korea Aerospace University, 76 Hanggongdaehak-ro Deogyang-gu, Goyang-si, Gyeonggi-do 10540, Korea; wklee@kau.ac.kr (W.-K.L.); taehwan.kim@kau.ac.kr (T.-H.K.); 3 Satrec Initiative, 21 Yuseong-daero 1628beon-gil Yuseong-gu, Daejeon 34054, Korea; jhpark@satreci.com

**Keywords:** video surveillance, video analytics, shadow detection, shadow removal, object extraction, regions of light sources, nighttime video, vertical histogram

## Abstract

Intelligent video surveillance systems detect pre-configured surveillance events through background modeling, foreground and object extraction, object tracking, and event detection. Shadow regions inside video frames sometimes appear as foreground objects, interfere with ensuing processes, and finally degrade the event detection performance of the systems. Conventional studies have mostly used intensity, color, texture, and geometric information to perform shadow detection in daytime video, but these methods lack the capability of removing shadows in nighttime video. In this paper, a novel shadow detection algorithm for nighttime video is proposed; this algorithm partitions each foreground object based on the object’s vertical histogram and screens out shadow objects by validating their orientations heading toward regions of light sources. From the experimental results, it can be seen that the proposed algorithm shows more than 93.8% shadow removal and 89.9% object extraction rates for nighttime video sequences, and the algorithm outperforms conventional shadow removal algorithms designed for daytime videos.

## 1. Introduction

Recently, video analytics has been widely deployed in various application areas including video surveillance, business intelligence, and the Internet of Things [1,2,3,4,5,6]. Especially in video surveillance systems, intelligent video analytics can reduce the cost of video monitoring and increase surveillance system performance by automatically analyzing video content to detect a variety of events such as intrusions, unattended objects, violence, fire, camera tamper attacks, and so on.

**Figure 1:**
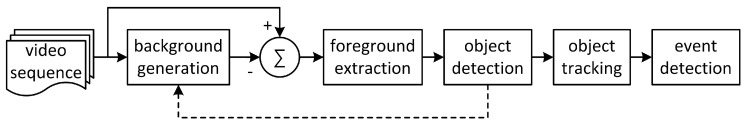
Video content analytics algorithm based on background subtraction.

Intelligent video analytics algorithms can be deployed in IP cameras or video analytics servers located in monitoring centers or cloud networks. A conventional video analytics algorithm based on background subtraction is shown in Figure 1 [2,5,6]. To extract foreground objects, background generation, binarization, and labeling are performed on input images. Finally, object tracking and predefined event detection are performed. Most conventional video analytics algorithms have used a background frame as a reference for comparison with a current frame [2,5,6]. The Gaussian mixture model (GMM) and the temporal averaging model are generally used for background generation [6,7]. For foreground extraction, background subtraction followed by binarization is used; this process compares the pixel difference between the background and the incoming frames with a certain threshold. Connected component labeling is used to obtain object information from the foreground pixels. Small labeled objects less than a certain threshold in size are considered to be noise blobs and are removed. Object tracking is performed on the labeled objects; this process relates the same object existing in successive frames. Event detection is the process of finding pre-configured events that have occurred in spatial and temporal directions using information such as the locations of labeled objects, the regions of interest (ROIs), the interactions among labeled objects, and the changed information of the objects.

**Figure 2:**
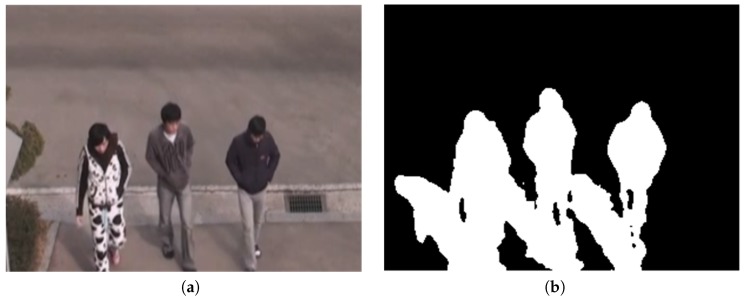
Foreground region extracted in a video content analytics algorithm: (**a**) Input video frame; (**b**) Foreground region extracted.

The performance of the event detection system mostly depends on the accuracy of foreground extraction, which is degraded by either foreground pixels absorbed into the background or shadow pixels detected as foreground. If the signal characteristics of foreground pixels are similar with those of the background pixels of the same positions, these foreground pixels can be considered as background. Also, it is highly probable that each shadow region will be extracted as foreground after background subtraction because the foreground intensity may be different from that of the background for the same position depending on the intensities of the illuminating light sources. Figure 2 shows an example of foreground extraction without shadow removal for a daytime video sequence. Three foreground objects and their shadow regions are merged into one large foreground object, which results in performance degradation in the ensuing object tracking and event detection stages. Therefore, to prevent performance degradation in video analytics algorithms, it is necessary to detect and remove shadow regions from extracted foreground regions.

Shadow regions are generally assumed to become darker than background regions, but they generally have similar characteristics in terms of chromaticity and texture with the background regions of the same position. Conventional shadow detection algorithms have used the similarities in color [8,9,10,11,12,13,14], geometric components [15,16,17,18], and texture [19,20,21,22,23] of shadow regions to separate these regions from foreground regions for daytime or indoor video sequences. Most recently, shadow region learning frameworks using multiple convolutional deep neural networks have been proposed for recovering images [24,25] and preprocessing video for surveillance systems [26].

Chromaticity-based shadow detection algorithms assume that background regions under shadow become darker but maintain their chromaticity; i.e., they exhibit color constancy. These algorithms choose color models of separate intensity and color components, such as normalized RGB [8], c1c2c3 [9], HSI [9], YUV [10] and HSV [11]. Although chromaticity-based methods are computationally inexpensive, they are susceptible to noise and are less effective in low saturated scenes; they often require explicit tuning of a large set of parameters for each scene [12,13,14].

Geometry-based shadow detection algorithms utilize the orientation and shape of the shadow regions, along with a knowledge of light sources, background surface condition, and object shapes such as those of vehicles [15,16] and pedestrians [17,18]. To distinguish shadow regions cast by pedestrians from foreground blobs generated by background subtraction, Hsieh [17] and Chen [18] assumed that human figures are posed vertically and that both human and shadow regions within a foreground blob are connected components. Hsieh [17] separated a human figure and its shadow region on a foreground blob using a line calculated from a histogram projection and the orientation of the blob, and refined the shadow region using the Gaussian shadow model based on orientation, mean intensity, and the center position of the shadow region. Chen identified linear boundaries separating human figures and their shadow regions using a 2-stage classifier trained with a multi-cue descriptor that included aspects of color, pixel location, and edge orientation representing the spatial constraint between human figures and shadows [18]. Although these algorithms can detect a shadow region for a single pedestrian with high accuracy under strong light sources, they cannot handle shadow regions disconnected from human figures and shadow regions of multiple pedestrians with occlusions.

Texture-based shadow detection algorithms utilize the fact that shadow regions keep most of their texture; using texture correlation, these algorithms compare the texture in the candidate shadow regions with that in the background. Various forms of texture correlation are proposed for shadow detection: gradient or edge correlation [19,20], orthogonal transform [21], SIFT [22], and Gabor filtering [23]. Although textures are robust to illumination changes, highly distinctive, and independent of colors, analysis using textures is computationally expensive.

Physics-based shadow detection algorithms use physics-based attenuation and color features to learn local or global shadow models [13,27,28,29]. Non-linear attenuation models of light sources are used to predict the color change of shadow regions in various illumination conditions [13,27]. To adapt to environmental changes, statistical learning of shadow pixels has been used to model the spectral properties of shadow pixels [13,27,28,29]. However, learning of local shadow models suffers from insufficient training data [27,28,29]. Also, these algorithms are still limited to handling objects with chromaticity similar to that of the background, and will require further performance improvement for practical use.

Due to the weakened signal strength of color and texture and increased noise power, these conventional algorithms have limitations in using color and texture features for shadow removal in nighttime video sequences. The conventional algorithms using the geometric characteristics of shadow [14,18,22] are only targeted at removing shadow regions of single isolated objects and cannot handle the interference of shadow regions in multiple object extraction.

Machine learning based shadow detection algorithms have proposed frameworks that automatically learns the most relevant features of shadow in a supervised manner using multiple convolutional deep neural networks [24,25,26]. These frameworks have strength in that there is no prior assumptions about the scene, the shadow properties, and the shape of objects. For these frameworks to be used for video pre-processing in video surveillance systems, further study is required to evaluate the cost for supervised training and the overall system complexity and to improve the shadow removal performance in various surveillance environments.

**Figure 3:**
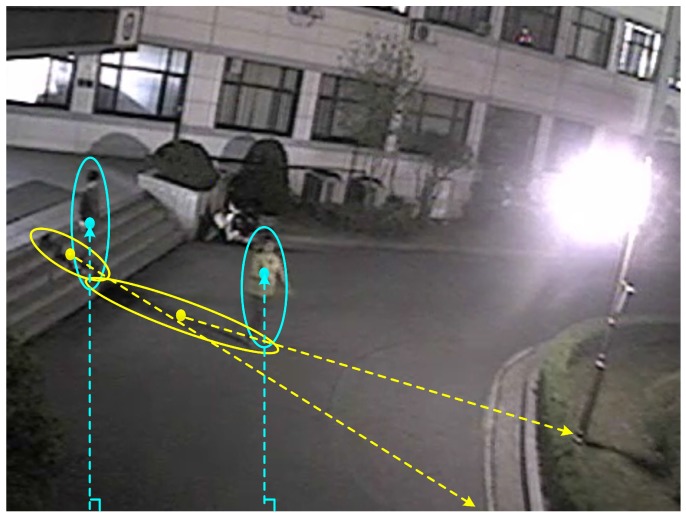
Shapes of objects and their shadow regions in nighttime video sequences.

In nighttime video sequences, as in the example shown in Figure 3, because the orientation of shadow region is determined by the displacement of an object from artificial light sources, the orientation of each object or shadow region can be used to detect shadow. In this paper, to remove shadow regions in nighttime video sequences, a novel shadow detection algorithm is proposed that partitions each foreground region into one or multiple partitioned objects based on object’s vertical histogram; the algorithm screens out shadow regions by validating the orientation of the partitioned object heading toward regions of light sources. The organization of this paper is as follows. In Section 2, a shadow detection algorithm based on the regions of light sources and shapes and orientations of foreground objects is proposed for object extraction in nighttime video sequences. In Section 3, experimental results for the proposed algorithm are presented and compared with results of conventional shadow detection algorithms. In Section 4, our conclusions and suggestions for further work are presented.

## 2. Shadow Removal Using Regions of Light Sources in Nighttime Video

The orientations of shadow regions can be argued to be strong features for shadow removal in nighttime video sequences. In this section, a novel shadow detection algorithm is proposed for nighttime video sequences; this algorithm uses the orientations of shadow regions toward the regions of light sources (RLS).

### 2.1. Overview of the Proposed Shadow Detection Algorithm

For shadow removal in nighttime video sequences, four assumptions are made concerning the shapes and orientations of objects and shadow regions. First, each target object and its shadow region can be matched to ellipses with high aspect ratio. Second, the orientation of each shadow region, the direction of the major axis of the ellipse matched to the shadow region, heads toward a region of a light source existing inside or outside the video frame. Third, the horizontal axis of the video frame is parallel to the ground plane, and the orientation of each target object is perpendicular to the ground plane. Finally, the orientation of each target object is different from that of its shadow. When a target object is located between a light source and a camera, the orientations of the target object and its shadow are in similar directions. Because cameras are usually installed in locations that avoid counter-light, the case of similar orientations is excluded in this study.

**Figure 4:**
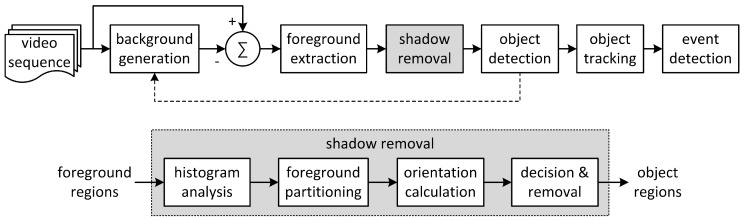
Video content analytics with the proposed shadow detection algorithm for nighttime video sequences.

Under these assumptions, each foreground object is partitioned based on its vertical histogram, and the matched ellipses of the partitioned objects are calculated. The orientation of each matched ellipse is used to validate each partitioned object as a shadow region. If the orientation heads toward the pre-configured regions of light sources, the corresponding partitioned object is classified as shadow and removed from the foreground region it belongs to. The video analytics system with the proposed shadow detection algorithm for nighttime video sequences is illustrated in Figure 4.

After background generation from the input video frames, binary foreground regions are extracted by background subtraction followed by median filtering for noise suppression. The proposed shadow detection algorithm is applied to the labeled foreground regions to provide final labeled object regions without shadow to the ensuing processes in the video analytics system. The proposed algorithm consists of histogram analysis, foreground partitioning, orientation calculation, and shadow decision and removal. By detecting abrupt changes in the vertical histogram inside each extracted foreground region, the foreground region is partitioned into one or multiple partitioned objects. For the calculation of the orientation of each partitioned object, that object is matched to an ellipse and the direction of the major axis of the matched ellipse, i.e., its orientation, is found. For each partitioned object, if its orientation heads toward the regions of light sources, it is classified as shadow and is used to make a shadow removal mask. Final object regions are obtained by masking the partitioned objects classified as shadow from the foreground regions.

### 2.2. Foreground Partitioning Based on Vertical Histogram

**Figure 5:**
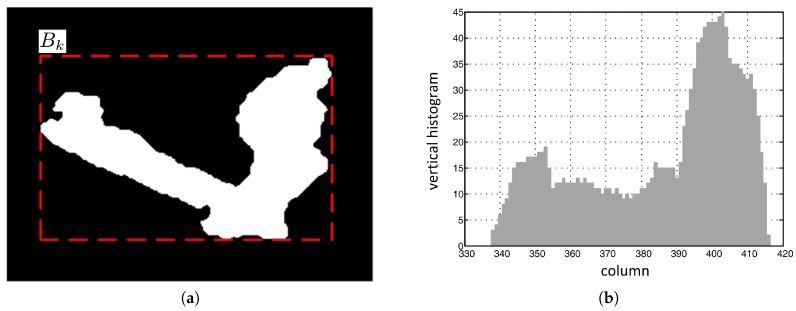
Vertical histogram of a foreground region: (**a**) A foreground region; (**b**) vertical histogram.

In nighttime video sequences, if the direction of a certain light is not similar to the orientation of a certain object, the object and its shadow appear as a merged foreground region that cannot be matched with a single ellipse, as shown in Figure 5a. In the bounding box enclosing the merged foreground region in a video frame, the vertical histogram in the shadow region is smaller than that in the object region. Therefore, in this section, a vertical histogram based foreground partitioning algorithm is proposed that uses this characteristic of vertical histograms in the merged foreground region to separate the shadow from the object.

The bounding box $B^{k}$ of the *k*th merged foreground region is represented in matrix form by its elements $\left\{b_{ij}^{k}\right\}$, of which the values are 0 or 1 for background and foreground pixels, respectively.

The vertical histogram of the *j*th column in the bounding box $B^{k}$ is defined as the number of foreground pixels per column, and is given as follows.

(1) $$\begin{array}{c} H_{j}^{k}=\sum_{i=0}^{h^{k}}b_{ij}^{k}, \end{array}$$

where $h^{k}$ represents the height of the bounding box $B^{k}$. Figure 5b shows the vertical histogram for the foreground object in Figure 5a.

After calculating the vertical histogram, the histogram difference between adjacent columns is compared with a given threshold to partition the foreground region $B^{k}$. Across the boundary between a foreground object and its shadow, the histogram difference is not always large enough for partitioning due to the various shapes of non-rigid foreground objects; this histogram difference is susceptible to noise at night. Also, the boundary is hard to define explicitly. Therefore, while scanning the columns from left to right or from right to left in a bounding box, the vertical histogram of the column that is *K* columns away from the current column is compared with that of the current column, the reference column, to aggregate enough of a histogram difference for partitioning. For histogram comparison, the number of columns that separate these two columns, *K*, depends on the resolution of the video frame; this number is 5 columns for *D1* resolution in this study. If there exists a column for which the vertical histogram is larger or smaller than that of the reference column by the amount of a certain threshold, the reference column is determined to be a partitioning column. A zero column vector is overwritten to the partitioning column to delineate one partitioned region from the other in the region $B^{k}$.

**Figure 6:**
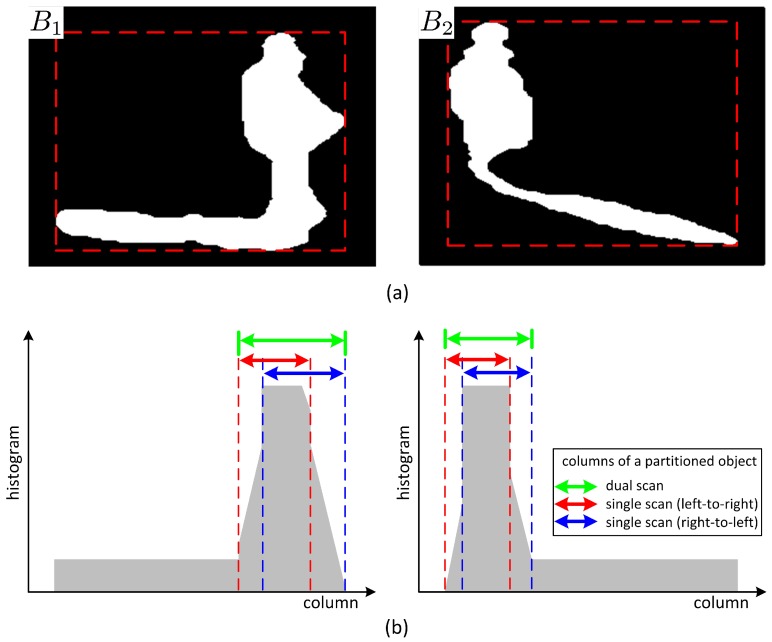
Partitioning of a foreground region by scanning the vertical histogram: (**a**) Foreground regions; (**b**) Foreground pixel histograms and the partitioned results for three scanning methods.

Shadow may exist either to the left or to the right of a foreground object, as shown in Figure 6a, depending on the location of a light source. Shadow may also exist between foreground objects. The shapes of foreground objects, mostly human bodies in this study, may vary over time while moving. For examples, due to movement of joints in arms, legs, or waists, the shape of a foreground object may be in the form of a normal cylinder, a cylinder with symmetric salience, or a cylinder with asymmetric salience. For a foreground object not in the form of a normal cylinder, thresholding the vertical histogram difference of adjacent columns may result in different partitioning results depending on the column scan directions. As shown in Figure 6, partitioning by comparison of vertical histogram only in a certain direction during column scan may result in an erosion of the salience from the opposite direction. This erosion of the salience may cause loss in the foreground object region. Therefore, double column scans in both directions are proposed to obtain candidates for partitioning column vectors. After double column scans, the partitioning column vectors far from the center of the cylindrical body are selected as the final partitioning column vectors.

For the calculation of a partitioned bounding box from an input bounding box, a foreground partitioning algorithm in a bounding box is proposed and is described in Algorithm 1. In the algorithm description, $W^{k}$, $0^{k}$, and $P^{k}$ represent the width, the zero column vector, and the partitioned bounding box of bounding box $B^{k}$, respectively. The partitioned bounding box is identical to the bounding box except for several partitioning columns overwritten by zero column vectors. The vectors $p_{j}^{k}$ and $b_{j}^{k}$ represent the *j*th column vectors in $P^{k}$ and $B^{k}$, respectively. TH represents the threshold for partitioning, and is constant over all video sequences. Although the variation in this threshold may slightly change the positions of the partitioning zero column vectors in the foreground region, this cannot greatly affect the shapes and pixel areas of partitioned objects and the centers of gravity of resulting foreground objects which are related to calculate the performance measures in Section 3.3.

**Figure 7:**
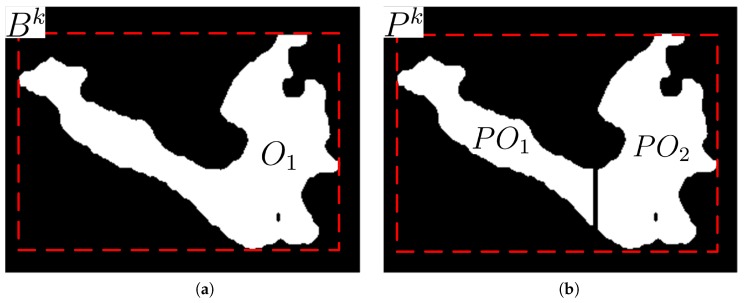
Foreground region partitioned into two smaller regions: (**a**) A foreground region extracted; (**b**) Partitioned regions after the double column scan partitioning.

Figure 7 shows the original and its partitioned regions in the bounding box and its partitioned bounding box, respectively. The partitioned regions are re-labeled as partitioned object $PO_{k}$ after the double column scan partitioning. If the number of pixels in a re-labeled object is less than $N_{obj}$, this object is treated as noise and removed from the object list.

**Algorithm 1:** PartitionBBox ($B^{k}$.)
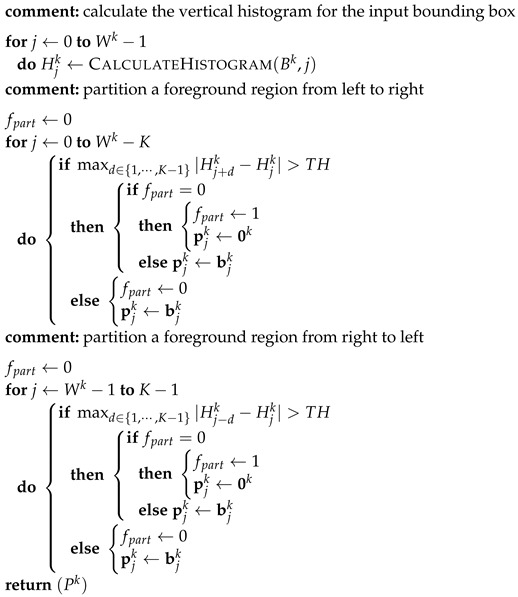


### 2.3. Calculation of the Direction of the Major Axis

In this study, every foreground object is assumed to be in the form of an ellipse and to have a matched virtual ellipse. The orientation of a partitioned object is defined as the direction of the major axis of the matched ellipse of the partitioned object. The center of the matched ellipse is defined as the centroid of the partitioned object. The direction can be found by rotating the partitioned object clockwise with the rotation axis at the centroid until the difference between the maximum and the minimum y-coordinates of the pixels in the rotated partitioned object is minimized.

The centroid of a partitioned object can be determined as follows.

(2) $$\begin{array}{c} \left(x_{c}^{PO_{k}},y_{c}^{PO_{k}}\right)=\left(\frac{\sum^{N_{k}}x_{i}^{PO_{k}}}{N_{k}},\frac{\sum^{N_{k}}y_{i}^{PO_{k}}}{N_{k}}\right), \end{array}$$

where $x_{i}^{PO_{k}}$, $y_{i}^{PO_{k}}$, and $N_{k}$ represent the *x*- and *y*- coordinates of the $i^{th}$ foreground pixel and the number of foreground pixels in the *k*th partitioned object, respectively.

**Figure 8:**
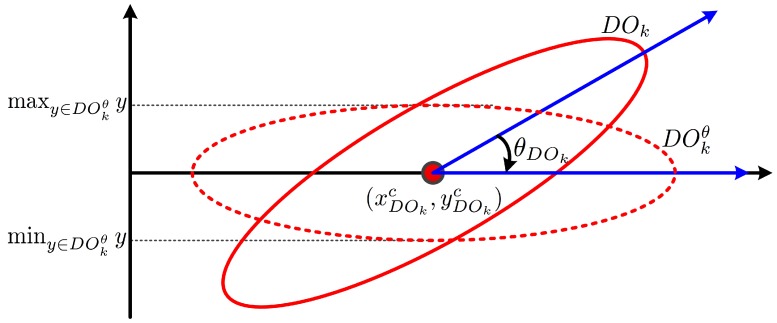
Finding the direction of the major axis of a matched ellipse.

**Figure 9:**
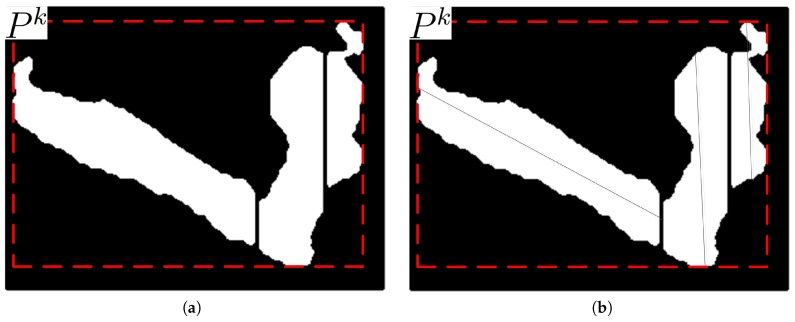
Calculation of the major axis of each partitioned object after vertical histogram analysis: (**a**) Partitioned objects; (**b**) Major axes of the virtual matched ellipses.

The angle between the major axis of the partitioned object and the horizontal axis, illustrated in Figure 8, is given as follows.

(3) $$\begin{array}{c} \theta^{PO_{k}}=\arg\min_{\theta }\left[\max_{y\in PO_{k}^{\theta }}y-\min_{y\in PO_{k}^{\theta }}y\right], \end{array}$$

where $PO_{k}^{\theta }$ represents the object from the partitioned object $PO_{k}$ rotated by the angle *θ* in a clockwise direction. Figure 9 shows the partitioned objects after vertical histogram analysis and after their major axes are calculated.

If the lower left corner of a video frame is set to the origin, the major axis can be represented by the following line equation.

(4) $$\begin{array}{c} l\left(x\right)=\tan\theta^{PO_{k}}\cdot \left(x-x_{c}^{PO_{k}}\right)+y_{c}^{PO_{k}}. \end{array}$$

### 2.4. Regions of Light Sources

**Figure 10:**
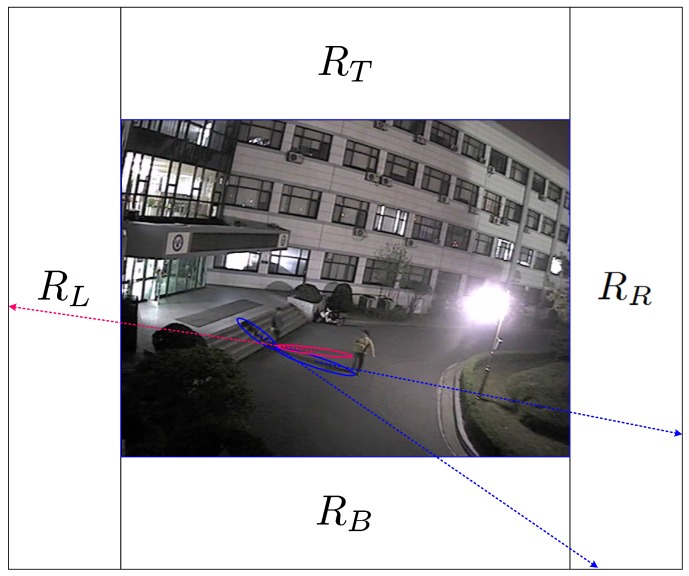
Possible regions of light sources for the proposed shadow detection algorithm.

Shadow regions in nighttime video sequences are formed by artificial light sources; their sizes and orientations are determined by their displacements from light sources. To estimate the orientation of each shadow, the locations of light sources should be known. Light sources in nighttime video sequences generally are not point sources, but ambient sources from buildings or explicit areas inside or outside the video frame, as shown in Figure 10. Because it is difficult to estimate the exact locations or areas of light sources, regions of light sources are configured manually outside the video frame, as shown in Figure 10.

Although light sources may exist inside the video frame, regions of light sources are configured on the left region $R_{L}$ or the right region $R_{R}$ outside the video frame by considering the orientations of the shadow regions formed inside the video frame. There may exist multiple RLS in $R_{L}$ or $R_{R}$, and the overall area of RLS in $R_{L}$ or $R_{R}$ may be equal to or less than those of $R_{L}$ and $R_{R}$.

In the proposed shadow detection algorithm, if the major axis of a partitioned object passes through the RLS, this partitioned object is considered to be a shadow. If light sources exist in the region $R_{B}$, the region of each foreground object overlaps with a significant portion of its shadow region. Although some saliences from a foreground object may exist due to shadow, there is little problem of a foreground region consisting of an object and its shadow being detected as a single object because the areas of the saliences are not very large. If light sources exist in the region $R_{T}$, it is difficult to use only the orientation of each partitioned object for shadow detection because the shadow region does not overlap the foreground object and its orientation is similar with that of the foreground object. Although this case requires other features such as color and texture as in daytime shadow removal, cameras are only rarely installed in such light source environments; rather, they are installed in environments that do not have counter-light. Therefore, in this study, as specified in Section 2.1, light sources are assumed to exist in the regions other than the region $R_{T}$.

### 2.5. Detection and Removal of Shadow Regions

For each partitioned object, if object orientation heads toward the RLS, it is classified as shadow. The shadow indicator for each partitioned object is given as follows.

(5) $$\begin{array}{c} S\left(PO_{k}\right)=\left\{\begin{array}{cc} 1, & \left(x,l(x)\right)\in R_{LS},\forall x\in R_{LS}^{X}\\ 0, & \mathrm{otherwise} \end{array}\right. \end{array}$$

where $R_{LS}^{X}$ represents the possible *x*-range of the RLS. The value of 1 represents a shadow region; 0 represents a foreground object.

The shadow indicator is used to make a shadow mask for a video frame; final foreground objects are obtained by masking the shadow regions from the input foreground regions.

## 3. Experimental Results

To evaluate the performance of the proposed algorithm, the video content analytics algorithm with the proposed method of shadow detection, shown in Figure 4, is implemented. A binary foreground image is obtained after background subtraction and thresholding; bounding boxes of foreground regions are calculated by labeling the image. The Gaussian mixture model [7] and contour labeling [30] are used for background modeling and for labeling of foreground pixels as regions, respectively.

The proposed shadow detection algorithm is fed with the foreground regions and their bounding boxes, obtained using the foreground extraction module in the video content analytics algorithm. The performance of the proposed algorithm in terms of both pixel and object levels is compared with those of conventional algorithms that adopt different features for shadow detection in daytime.

### 3.1. Experimental Environments

To evaluate the performance of the proposed shadow detection algorithm, six nighttime and two daytime surveillance video sequences, listed in Table 1, were used for the experiments. The one daytime and six nighttime sequences were captured on a university campus. The one daytime sequence is taken from the PETS2001 data-set [31]. All the sequences are in D1 (720 × 480) resolution at 30 Hz. In all the nighttime sequences, there exist shadow regions generated by light sources inside or outside the video frames. The interference *single* is a video sequence with a single foreground object in every video frame in which there is no interference from other foreground objects or their shadow regions. The interference *multiple* is a video sequence with multiple foreground objects in most of the video frames in which there exists interference from neighboring foreground objects or their shadow regions. Although the proposed algorithm attempts to detect and remove shadow regions in nighttime video sequences, the performance for two daytime video sequences is measured for performance reference.

### 3.2. Configuration of RLS

For each video sequence, RLS are configured outside the video frames based on the locations of light sources generating shadow. Although each partitioned object can be matched to an ellipse, the orientation of the matched ellipse may be inaccurate due to imperfect extraction of foreground regions. Also, the ground where foreground objects exist may incline to one side, which modulates the orientation of each matched ellipse with the grade of the slope. Although estimation errors in the orientations of matched ellipses are hard to predict for these cases, the errors are not so large as to considerably change the orientations. Therefore, only the existence of left or right light sources is considered for shadow removal; this is done simply by configuring entire left or right regions, $R_{L}$ or $R_{R}$, as RLS, as shown in Figure 10. For the experiment using the D1 video sequences, the width and the height of each $R_{L}$ or $R_{R}$ are set to 200 and 720 pixels, respectively. The column *RLS* in Table 1 shows the configuration of RLS for each sequence.

### 3.3. Measures for Performance Evaluation

For performance evaluation, the ground truths are generated by painting foreground objects by hand; these objects are compared with the final foreground objects generated by the proposed shadow detection algorithm. Because the proposed algorithm is fed by the foreground regions from the foreground extraction module, consisting of background subtraction and thresholding, only the extracted region of each foreground is considered as the ground truth. Foreground regions not extracted because their statistical characteristics are similar to those of the background are not included in the ground truth.

Two performance measures for shadow detection and the ensuing process in the video analytics system are defined: shadow removal rate and object extraction rate. Shadow removal rate is defined as the measure of the performance of shadow removal from the extracted foreground regions at the pixel level, as follows.

(6) $$\begin{array}{c} \eta =\frac{TP_{s}}{TP_{s}+FN_{s}}, \end{array}$$

where $TP_{s}$ and $FN_{s}$ represent the number of shadow pixels determined to be shadow and the number of shadow pixels determined to be foreground in the extracted foreground regions, respectively.

For shadow detection in video content analytics systems, it is important to measure how shadow removal affects the performance of object extraction. In the i-LIDS guide [32], the correctness of object extraction is judged by the distance between the object extracted and its ground truth. If the Euclidean distance between the centers of gravity of the object extracted and its ground truth is less than a certain threshold, this object is determined to be the correctly extracted object ($TP_{o}$). Otherwise, this object is determined to be an interfered object ($FN_{o}$). The threshold for decision is defined as half the width of the bounding box of the ground truth. To measure the performance of object extraction on the object level before and after shadow removal, object extraction rate is defined as follows.

(7) $$\begin{array}{c} \zeta_{o}=\frac{TP_{o}}{TP_{o}+FN_{o}}, \end{array}$$

where $TP_{o}$ and $FN_{o}$ represent the number of objects correctly extracted and the number of objects not correctly extracted, respectively.

### 3.4. Step-Wise Results for the Proposed Shadow Detection Algorithm

**Figure 11:**
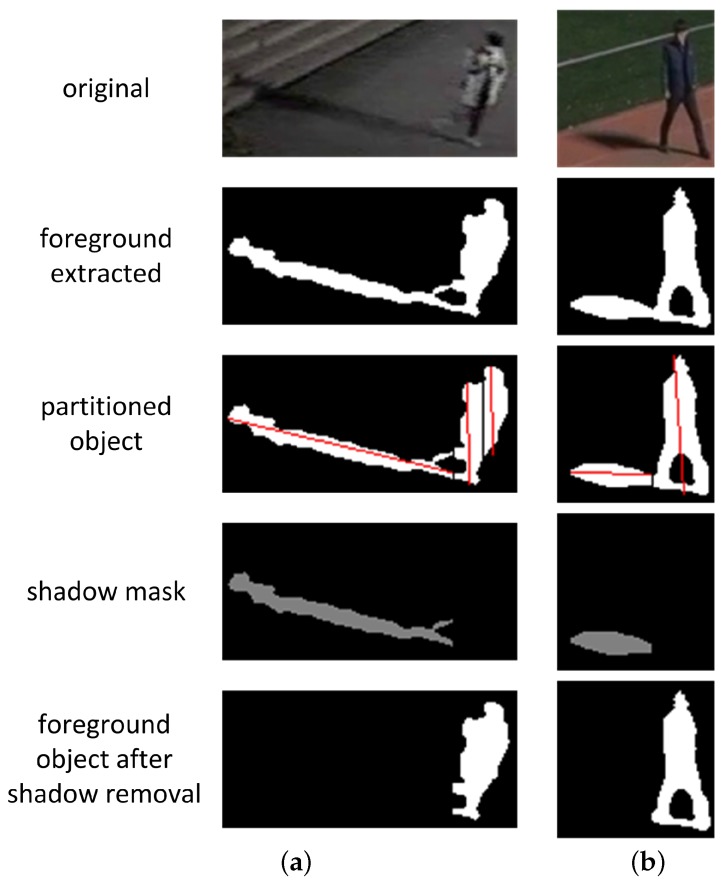
Results in each step in the proposed shadow detection algorithm: (**a**) Sequence S5, frame 1280; (**b**) Sequence S3, frame 826.

Figure 11 shows the step-wise processing results for two foreground regions with shadow; these regions are extracted from different sequences. The shadow regions in both the foreground regions are discernible from the background, and the extracted foreground regions include shadow regions. The foreground regions in Figure 11a,b are partitioned into three and two partitioned objects, respectively. For both examples, the partitioned objects, of which the orientations head toward the RLS outside the video frames, are determined to be shadow. The other partitioned objects, the final foreground objects, have different orientations from the shadow regions; this direction is upwards in normal cases.

#### 3.4.1. Shadow Removal for a Single Object

For a single object, there is no possibility for each shadow region to interfere with other objects. However, to prevent false alarms in event detection, shadow removal for a single object is also required for video content analytics because a shadow region is sometimes separated from its host object and detected as a new object; or, it intrudes on regions of interest while its host does not.

**Figure 12:**
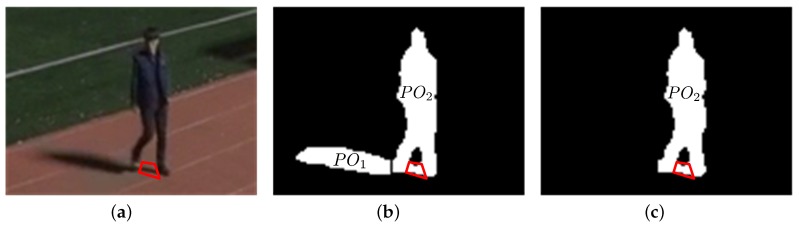
Shadow removal result 1 for a foreground object (120 × 90 pixels from sequence S3): (**a**) A foreground object; (**b**) Partitioned objects; (**c**) A foreground object after shadow removal.

**Figure 13:**
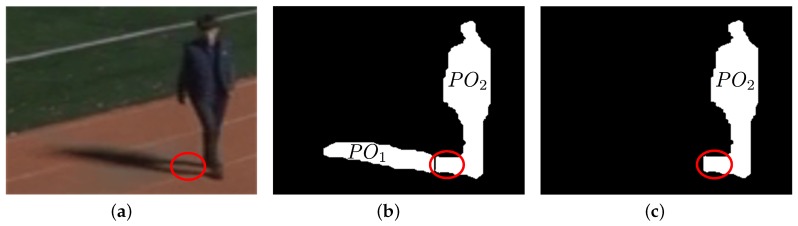
Shadow removal result 2 for a foreground object (87 × 84 pixels from sequence S3): (**a**) A foreground object; (**b**) Partitioned objects; (**c**) A foreground object after shadow removal.

Figure 12 and Figure 13 show the shadow removal results for a single object isolated or separated from other objects. During the partitioning process, as shown in Figure 12b and Figure 13b, zero column vectors are inserted into the boundary columns of a foreground object with a large vertical histogram difference with its neighboring columns. However, this insertion does not erode the boundary columns of the foreground object, as can be seen in Figure 12c and Figure 13c, because only the partitioned shadow is used to generate the object’s shadow removal mask.

Although the final foreground objects after shadow removal still include several shadow pixels, as can be seen in the red enclosed regions in Figure 12c and Figure 13c, it can be argued that the remaining shadow pixels do not interfere with the ensuing object tracking process because these pixels are located right underneath the foreground objects and the number of these pixels is not large compared with the number of pixels of the foreground object. For applications other than object tracking, these shadow pixels underneath foreground objects can be removed if other features of shadow such as color and texture are used.

#### 3.4.2. Shadow Detection for Multiple Objects

**Figure 14:**
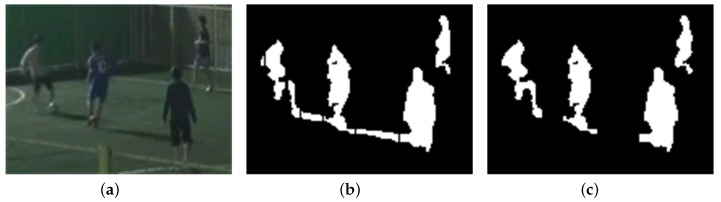
Shadow removal result 3 for multiple foreground objects (140 × 105 pixels from sequence S4): (**a**) Foreground objects; (**b**) Partitioned objects; (**c**) Foreground objects after shadow removal.

**Figure 15:**
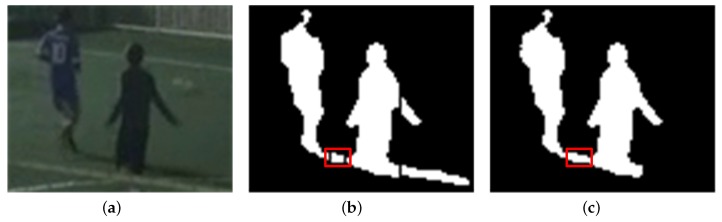
Shadow removal result 4 for multiple foreground objects (140 × 105 pixels from sequence S4): (**a**) Foreground objects; (**b**) Partitioned objects; (**c**) Foreground objects after shadow removal.

For multiple objects, some objects are sometimes interfered with by shadow regions of other objects if the objects are close enough to be connected by shadow regions. The proposed algorithm can also remove shadow regions between neighboring objects by partitioning foreground regions based on vertical histogram comparison and validation of the orientations of partitioned objects. Figure 14 and Figure 15 show the shadow detection and removal results for sequence S4 with multiple objects.

Three objects in Figure 14b are connected to their neighboring objects via shadow regions that exist among them before partitioning. Most of the shadow regions between objects head toward the RLS and are removed from the foreground in Figure 14c. However, if foreground objects are very close to each other, the shadow regions between them, as shown in Figure 15, are sometimes not removed because they are too small to have dominant orientations toward the RLS.

**Figure 16:**
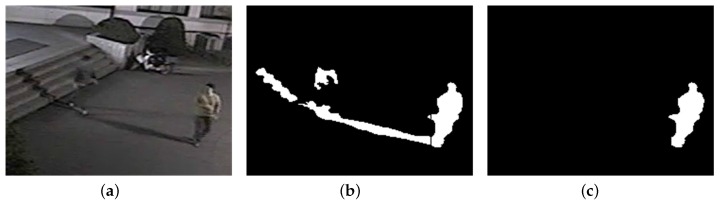
Shadow removal result 5 for a foreground object (240 × 180 pixels from sequence S2): (**a**) Foreground objects; (**b**) Partitioned objects (**c**) Foreground object after shadow removal.

Fragmented or incomplete foreground regions due to imperfect foreground extraction in the video analytics system may be detected as shadow regions if their orientations accidentally head toward the RLS. Figure 16 shows the shadow removal result for a video frame with an incomplete foreground region: the incomplete foreground region in Figure 16b disappeared in Figure 16c because its orientation was determined to head toward the RLS.

These problems of small shadow regions merged with foreground objects and the removal of incompletely extracted foreground regions can be resolved by applying features used in daytime shadow removal such as color and texture or by using object tracking information; these subjects are not considered in this study and are left for further study.

### 3.5. Performance Comparison

#### 3.5.1. Output Images

**Figure 17:**
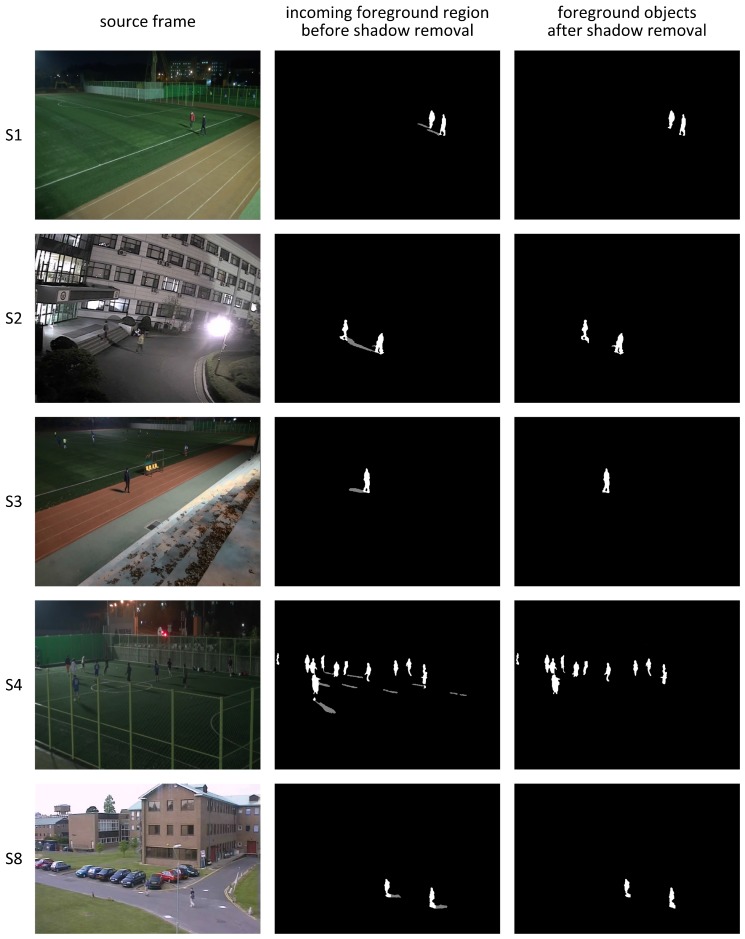
Shadow removal results for various nighttime and one daytime video sequences.

Figure 17 shows sample video frames and their processing results when using the proposed shadow detection algorithm for the test video sequences. Most of the shadow regions in the extracted foreground regions are successfully removed by the proposed algorithm, such that they do not interfere with foreground objects.

#### 3.5.2. Performance Comparison in Terms of Pixel and Object Levels

Using the shadow removal rate in Equation (6) and object extraction rate in Equation (7), the performance of shadow removal on the pixel level and the performance of object extraction before and after shadow removal are measured for the proposed and for five conventional algorithms. These conventional algorithms are based on features such as chromaticity (Chr) [11], geometry (Geo) [17], physics (Phy) [13], small region texture (srTex) [23], and large region texture (lrTex) [12]. The C++ implementations of these algorithms by Sanin et al. were used for the experiments [12]. The object extraction performance before shadow removal (BSR) was also measured for comparison.

Table 2 shows the shadow removal and object extraction rates before and after shadow removal for six nighttime and two daytime video sequences. Although the chromaticity based method shows an average shadow removal rate for nighttime video higher than those of the other conventional algorithms, its average object extraction rate is the lowest among the algorithms and below that of BSR. The average object extraction rates of the other conventional algorithms for nighttime video are slightly higher than that of BSR; this means that the conventional algorithms cannot be acceptable for video analytics systems. Although the conventional algorithms showed a performance enhancement for some of the nighttime sequences, they failed to enhance the performance for other nighttime sequences. None of them succeeded in enhancing the performance for every sequence. Especially, the chromaticity based algorithm showed its weakness at shadow removal and object extraction in nighttime video, because the chromaticity components are more weakened at night than are the other features, and the chromaticity components of the background and the foreground objects cannot be differentiated easily. For every conventional algorithm, a foreground object is sometimes found to be split into multiple small foreground blobs because the features of this algorithm for shadow removal in daytime sequences cannot efficiently differentiate shadow and object regions in nighttime sequences. For the calculation of object extraction rate in this case, the largest of the split blobs from a foreground object is treated as the extracted object.

The proposed algorithm shows a 93.8% shadow removal rate and a more than 40.0% enhancement over BSR in average object extraction rate for nighttime video. The object extraction rate of the proposed algorithm for sequence S4 cannot be acceptable for video analytics. The performance enhancement for sequence S4 is limited because the objects in the scene are quite close to each other, close enough to disturb the object extraction process; the front fence sometimes separates a single object into several smaller blobs, and some objects in the video frames of sequence S4 are less illuminated by light sources.

For daytime video sequences, all the algorithms showed better shadow removal rates than they did for nighttime video. Especially, the physics based algorithm and the proposed algorithm are superior to the others in object extraction rate.

#### 3.5.3. Shadow Removal Performance for Multiple Objects

For accurate object tracking, merge and separation of multiple objects should be handled appropriately. Shadow regions between neighboring objects may cause merging of neighboring objects and result in the loss of object information. If multiple objects are connected by shadow, these objects are usually labeled as a single object, losing their object information.

The shadow removal performance for multiple objects connected by shadow is evaluated for the proposed algorithm. Among the multiple object sequences in Table 1, only sequences S1, S2, and S4 have multiple objects connected by shadow. Table 3 shows the removal rate for object merged by shadow before and after applying the proposed shadow detection algorithm. For the multiple objects in sequences S1 and S2, after applying the proposed algorithm, no object merger due to interference of the shadow regions occurred because the proposed algorithm can detect and remove shadow regions among objects. For the multiple objects in sequence S4, while more than 70% of object mergers due to shadow were removed, there still existed object merger by shadow due to the relatively lower illumination and complex scene contents of the sports theme. The close distance between neighboring objects may cause the orientation of the shadow regions to be arbitrary, such that these regions sometimes cannot be removed using only the proposed algorithm.

It can be shown that the proposed algorithm, by removing shadow between neighboring objects, can prevent performance degradation due to object merger by shadow in normal nighttime video surveillance sequences. Although the proposed algorithm has a limitation in removing shadow pixels underneath objects, this limitation does not cause object merging or performance degradation in object tracking. However, for complex nighttime video sequences, further study to consider texture and color components together with the orientation of the partitioned object will be required to prevent performance degradation.

## 4. Conclusions

In this paper, a novel shadow detection algorithm is proposed for object extraction in nighttime video sequences; this algorithm validates the possible orientations of shadow toward the region of light sources. Each extracted foreground region is partitioned by double scan based vertical histogram change detection, each partitioned object is matched to an ellipse, and the orientation of the matched ellipse is used for the validation of the partitioned object as shadow. The proposed algorithm is shown to quite accurately remove shadow in nighttime video sequences with a single object or multiple objects, with a 93.8% shadow removal rate; this algorithm improves the object extraction performance by suppressing the interference among neighboring objects caused by their shadow regions.

The proposed shadow detection algorithm, combined with a conventional daytime shadow detection algorithm, can be used around the clock in intelligent video surveillance systems to detect pre-configured surveillance events based on object extraction.

The proposed algorithm has a limitation in handling small partitioned objects, such as small shadow regions between objects and incomplete foreground regions, of which the orientations are not explicitly discernible. This limitation can be resolved by applying features used in daytime shadow removal such as color and texture or by using object tracking information. The comparative study with machine-learning approach is also required for various video surveillance environments. These topics will be left for further study.

## Figures and Tables

**Table 1:** Test video sequences for shadow detection.

Seq. Number	Length (Frames)	Location	Time	Interference	*RLS*
1	300	a soccer field	night	*multiple*	$R_{R}$
2	200	an entrance of a building	night	*multiple*	$R_{L}\cup R_{R}$
3	200	a running track	night	*single*	$R_{R}$
4	300	a futsal field	night	*multiple*	$R_{L}$
5	1030	an entrance of a building	night	*single*	$R_{L}\cup R_{R}$
6	210	an entrance of a building	night	*single*	$R_{L}\cup R_{R}$
7	490	a crossroad	day	*multiple*	$R_{R}$
8	200	a forked road	day	*multiple*	$R_{L}$

**Table 2:** Performance comparison in pixel and object levels.

Sequence		BSR	Proposed		Chr		Geo		Phy		srTex		lrTex	
		$\zeta_{o}$	*η*	$\zeta_{o}$	*η*	$\zeta_{o}$	*η*	$\zeta_{o}$	*η*	$\zeta_{o}$	*η*	$\zeta_{o}$	*η*	$\zeta_{o}$
night	S1	79.9	90.6	98.9	36.5	25.1	53.4	75.0	0.1	74.8	13.5	81.2	6.0	75.0
	S2	16.9	96.0	93.4	88.1	34.9	62.3	50.0	7.0	21.2	51.1	79.0	11.0	34.9
	S3	35.8	88.7	99.0	80.1	19.4	74.0	36.8	0.5	36.3	34.3	16.9	16.3	43.3
	S4	53.5	98.0	57.7	47.6	22.7	44.8	43.1	11.2	47.4	45.2	37.7	18.0	49.2
	S5	53.4	94.9	92.4	85.1	57.5	60.2	75.3	4.1	57.3	37.1	51.4	4.7	59.7
	S6	52.1	94.4	98.1	86.0	35.1	71.9	38.4	8.0	70.1	51.3	87.7	51.6	71.1
	average	48.6	93.8	89.9	70.5	32.4	61.1	53.1	5.2	51.2	38.7	59.0	18.0	55.5
day	S7	84.8	93.8	97.1	96.2	60.4	67.5	49.3	69.9	95.6	89.0	85.2	92.3	81.5
	S8	35.8	80.2	98.3	88.8	62.5	67.6	63.9	60.7	95.5	69.7	81.1	13.5	50.1

**Table 3:** Removal rate of merged objects by shadow.

	Merged Objects by Shadow		Removal Rate of Merged Objects by Shadow (%)
	before	after	
S1	12	0	100.0
S2	59	0	100.0
S4	55	16	70.9
total	126	16	87.3

## Abbreviations

The following abbreviations are used in this manuscript:

RLS	regions of light sources
GMM	Gaussian mixture model
